# The Role of Exosomal microRNA in Cancer Drug Resistance

**DOI:** 10.3389/fonc.2020.00472

**Published:** 2020-04-07

**Authors:** Qiao-ru Guo, Hui Wang, Ying-da Yan, Yun Liu, Chao-yue Su, Hu-biao Chen, Yan-yan Yan, Rameshwar Adhikari, Qiang Wu, Jian-ye Zhang

**Affiliations:** ^1^Guangdong Provincial Key Laboratory of Molecular Target & Clinical Pharmacology, School of Pharmaceutical Sciences and the Fifth Affiliated Hospital, Guangzhou Medical University, Guangzhou, China; ^2^Key Laboratory of Tropical Translational Medicine of Ministry of Education, Hainan Medical University, Haikou, China; ^3^Guangzhou Institute of Pediatrics/Guangzhou Women and Children's Medical Center, Guangzhou Medical University, Guangzhou, China; ^4^School of Chinese Medicine, Hong Kong Baptist University, Hong Kong, China; ^5^Collaborative Innovation Center for Cancer, Institute of Respiratory and Occupational Diseases, Medical College, Shanxi Datong University, Datong, China; ^6^Research Centre for Applied Science and Technology, Tribhuvan University, Kirtipur, Nepal; ^7^Key Laboratory of Emergency and Trauma of Ministry of Education, School of Tropical Medicine and Laboratory Medicine, Hainan Medical University, Haikou, China

**Keywords:** exosome, miRNA, cancer, drug resistance, mechanism

## Abstract

Exosomes affect the initiation and progression of cancers. In the tumor microenvironment, not only cancer cells, but also fibroblasts and immunocytes secrete exosomes. Exosomes act as a communicator between cells by transferring different cargos and microRNAs (miRNAs). Drug resistance is one of the critical factors affecting therapeutic effect in the course of cancer treatment. The currently known mechanisms of drug resistance include drug efflux, alterations in drug metabolism, DNA damage repair, alterations of energy programming, cancer stem cells and epigenetic changes. Many studies have shown that miRNA carried by exosomes is closely associated with the development of drug resistance mediated by the above-mentioned mechanisms. This review article will discuss how exosomal miRNAs regulate the drug resistance.

## Introduction

Chemotherapy, radiotherapy, surgery, and targeted therapy are important modalities of cancer treatment. However, the emergence of drug resistance leads to dismal prognosis in cancer patients. As an emerging therapeutic target and diagnostic biomarker, exosomal miRNAs play vital roles in tumor invasion, metastasis and progression. Studies have found that the occurrence and development of drug resistance is closely related to miRNA carried by exosomes (1–5). In this review article we discuss several classic mechanisms that exosomal miRNA involves in drug resistance. Also, we summarize the role of exosomal miRNA mediated drug resistance in different types of cancers.

## The Biogenesis of Exosome and miRNA

Exosomes are extracellular vesicles (EV) with the size ranges between 30 and 100 nm. Exosomes are released to the extracellular environment after the fusion of the multivesicular body (MVB) or late endosomes with the plasma membrane (6). It was first described in 1983 as “seems to be akin to reverse endocytosis” (7), and has gradually recognized an important factor in oncology research (8). Many studies have shown that exosome can promote the intercellular communication (9) by transferring varieties of cargos, such as nucleic acid, proteins and metabolites (10–16).

**Figure 1:**
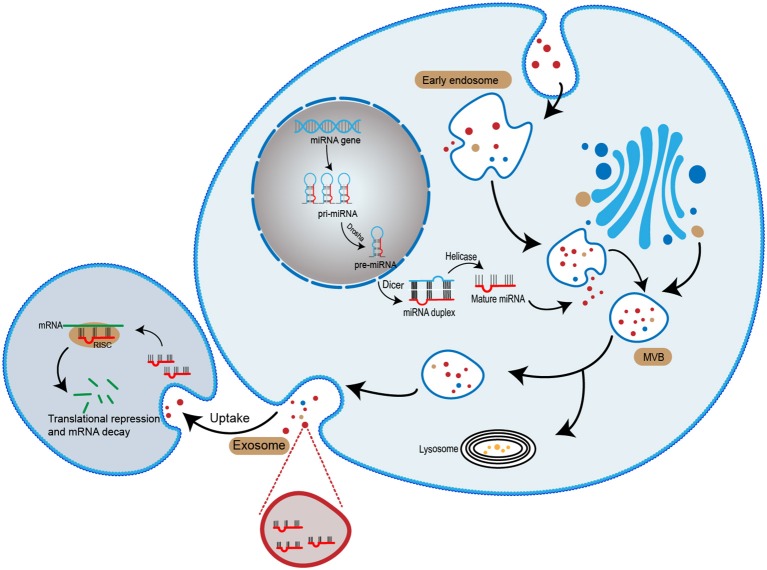
The biogenesis of exosomal miRNA. miRNA genes are transcribed into primary miRNA (pri-miRNA). The pri-miRNA is cleaved by Dorsha complex to produce precursor miRNA (pre-miRNA) and exported into the cytoplasma. Subsequently, pre-miRNA is processed by Dicer complex to become mature miRNA. Mature miRNA is sorted into exosomes and released to the extracellular space. With the recipient cell uptake exosomes, the miRNA entered the cell and mediates gene suppression by targeted translational repression and mRNA degradation.

microRNA (miRNA) is an important cargo that delivered by exosomes (17). miRNAs usually consist of 19–25 nucleotides. It can regulate post-transcriptional silence of target genes. Following the transcription of miRNA gene, a small hairpin-shaped RNA called pre-miRNA is generated. The pre-miRNA is exported into cytoplasm and processed by Dicer, a kind of RNase III-type endonuclease, and subsequently releases a small RNA duplex (18, 19). The RNA duplex will unwind after loading onto an Argonaute (AGO) protein and forming RNA-induced silencing complex (RISC) (20). Once binding to a RISC, the miRNA is complementary pairing with the mRNA. Depending on whether miRNA and mRNA are fully bind, two different mechanisms occur: (1) mRNA specifies cleavage if miRNA is sufficiently complementary to mRNA; (2) the productive translation is inhibited when miRNA is insufficiently complementary to mRNA (21, 22). Therefore, miRNA can regulate various physiological and pathological activities (Figure 1).

## The Current Understanding of Cancer Drug Resistance

Cancer drug resistance can be divided into intrinsic and acquired resistance. Intrinsic resistance occurs before receiving therapy, which limits the use of anticancer drugs. Acquired resistance may develop during treatment even if some drugs have anticancer effects at the early stage (23). Drug resistance seriously impacts the effectiveness of chemotherapy and molecular targeted therapies, ultimately leading dismal prognosis and tumor relapse.

Because of genomic instability, tumors may include a diverse collection of cells that possess different sensitivity to treatment (24). The positive selection of drug-resistant tumor subpopulation causes drug resistance. Therefore, accurate assessment of tumor heterogeneity is important to address drug resistance (25). The application of high-throughput screening technology facilitates the identification of genotype and helps predict drug response, providing convenience to individual therapy. In the past few years, microfluidic chips show tremendous promise in the study of tumor heterogeneity and the establishment of preclinical models (26). Grosselin et al. (27) set up a high-throughput droplet microfluidics platform. On this platform, the single cell chromatin landscapes of thousand cells can be profiled. They used the patient-derived xenograft models of acquired resistance to chemotherapy and target therapies in breast cancer and found a common chromatin signature between drug-sensitive and resistant cells (27). This technique paves the way to study the role of chromatin heterogeneity.

The limited cancer models hinder the clinical prediction of drug efficacy. Therefore, it is urgent to establish more reasonable, advanced and high-throughput cancer models to deal with drug resistance and explore the underlying factor of heterogeneous patient responses. Gao et al. (28) established about 1,000 patient-derived tumor xenograft models (PDXs) with a diverse set of driver mutation using high-throughput screening technology. It has been demonstrated that these PDXs have the potential to predict patient response to targeted therapies and perform *in vivo* compound screens (28). Furthermore, a 3D model of tumor tissue made up of numerous different cell types can better mimic tumor microenvironment and provide the similar information about clinical response. Kather et al. developed a 3D model of tumor tissue which reproduced key features of colorectal cancer (CRC) and based on the individual patient data, yielding *in silico* tumor explant (29).

Combinations of drugs are also the effective way to overcome or bypass drug resistance (30). Epidermal growth factor receptor tyrosine kinase inhibitor (EGFR TKI) is beneficial for the treatment of non-small cell lung cancer with EGFR mutation (31). However, after treatment with EGFR TKI for 10–14 months, the efficacy declines (32), the primary and acquired drug resistance limits their clinical benefit (33). To combat resistance, in addition to developing new drugs, drugs combinations through a so-called bypass signaling mechanism, is an excellent choice (34). In addition, nanomedicine approach can be used to encapsulate and co-delivery drugs in specific materials to improve their bioavailability and thus overcome drug resistance (35, 36). The application of high-throughput drug screening can identify the effective drug combination regimens. Using high-throughput screening technology, researchers identified that potassium antimony tartrate in combination with topotecan can significantly enhance the sensitivity of non-small cell lung cancer and colorectal cancer to *cis*-diamminedichloroplatinum/cisplatin (CDDP). It was found that topotecan impairs the ability to repair CDDP-induced DNA damage (37). DNA damage repair is a classic mechanism by which cells develop drug resistance, as detailed later in this article.

Cancer biomarkers are present in tumor tissue or serum that help to detect cancers in their early stage, simplified the prognosis of cancer development (38). Cancer biomarkers help stratify patients to receive specific therapeutics. Biomarker could be DNA, mRNA, protein and various cellular metabolites (39). Over the past years, many advances have been made in the detection and evaluation of cancer biomarkers (40–44). In the next section, we will detail the development of exosomal miRNA as cancer biomarkers.

## Exosomal miRNA as a Regulator and Biomarker in Cancer

Exosomal miRNA is involved in the proliferation, invasion, migration and drug resistance of various cancers. Therefore, exosomal miRNA has the potential to be the biomarkers for cancer diagnosis and treatment. In Table 1, we summarize some recent researches on various exosomal miRNA as regulators and biomarkers in various cancers.

**Table 1:** Exosomal miRNA as regulators and biomarkers in different cancers.

**Cancer type**	**Exosomal miRNA**	**References**
Breast cancer	miR-9; miR-222; miR-105; miR-10b; miR-122; miR-1246	(45–50)
Colon cancer	miR-146a-5p; miR-200b; miR-193a; miR-125-3p; miR-25-3p; miR-27a; miR-130a	(14, 51–55)
Gastric cancer	miR-139; miR-130a; miR-21; miR-423-5p; miR-451; miR-10b-5p; miR-195-5p; miR-20a-3p; miR-296-5p	(4, 16, 56–59)
Lung cancer	miR-23a; miR-126; miR-96; miR-222-3p; let-7a-5p; miR16; miR-322; miR-497; miR-17	(60–65)
Liver cancer	miR-1237-3p; miR-335; miR-320a; miR-103; miR-18a; miR-221; miR-222; miR-224; miR-101; miR-106b; miR-122; miR-195	(66–70)
Ovarian cancer	Let-7; miR-200; miR-29a-3p; miR-21-5p; miR-205; miR-145; miR-200c; miR-940; miR-6126; miR-1246; miR-100; miR-320; miR-23a	(12, 71–77)
Pancreatic cancer	miR-21; miR-155; miR-365; miR-1231; miR-155; miR-301a; miR-1246; miR-4644; miR-3976; miR-4306	(78–83)
Prostate cancer	miR-1290; miR-375; miR-21-5p; miR-196a-5p; miR-501-3p; miR-1246	(84–87)
Oral cancer	miR-382-5p; miR-1246; miR-21; miR-34a-5p	(88–91)
Nasopharynx cancer	miR-23a; miR-24-3p; miR-9	(92–94)

Breast cancer (BC) is a highly prevalent cancer and the second leading cause of cancer-related death among women (95, 96). MiR-9 is a classic miRNA in cancer development. Baroni et al. indicated that exosomal miR-9 has the ability to induce human breast fibroblasts to have cancer associated fibroblasts (CAFs)-like properties (45). Exosome transferring miR-222 can promote BC cells migration and invasion by activating Nuclear factor-κB (NF-κB) (46). Exosomal miRNA also regulate tumor growth by influencing the metabolic reprogramming of BC cells. Yan et al. suggested that BC cells secrete exosomal miR-105 to promote tumor growth through the regulation of metabolic reprogramming in stromal cells (47).

Exosomal miRNA regulates tumor growth in other cancers as well. In colorectal cancer (CRC), transforming growth factor-beta (TGF-β) significantly contributes to the upregulation of exosome-meditated miR-200b, which promotes colorectal cancer cell proliferation by suppressing the expression of p27 in target cells. (51). Li et al. demonstrated that the absence of exosomal miR-148b derived from CAFs is the cause of invasion and metastasis in endometrial cancer (97). In lung cancer, Wu et al. indicated that exosomal miR-96 is associated with proliferation, migration and drug resistance by directly binding to wild-type LMO7 gene (60).

The expression of some exosomal miRNA in cancers is specific. The specificity enables exosomal miRNA to become cancer biomarkers. Sohn et al. proposed that serum exosomal miRNAs have the potential to become novel biomarkers for hepatocellular carcinoma (66). Moreover, Huang et al. extracted serum-derived exosome from patients with gastric non-cardia adenocarcinoma and detected the expression of miRNA. They identified the expression of miR-195-5p, miR-20a-3p, and miR-196-5p in exosomes and found that these miRNAs significantly increased. This finding provided a reference for clinical application and diagnosis using exosomal miRNAs (56).

## The Mechanism of Cancer Drug Resistance With Exosomal miRNA

**Figure 2:**
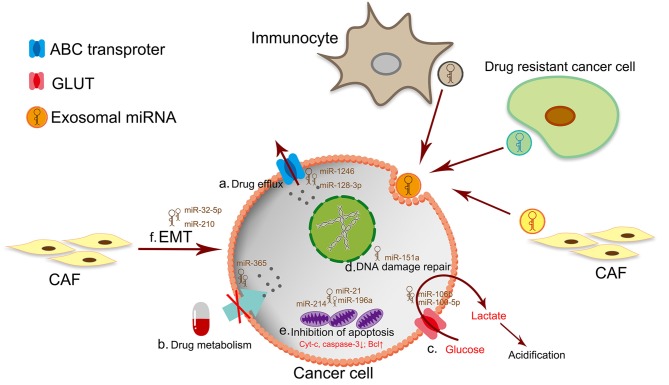
The mechanism of drug resistance in cancer cells. a. Drug efflux by the ATP-binding cassette (ABC) transporter superfamily. b. The dysfunction of drug metabolism by drug inactivation or lack of drug activation. c. The excessive production of lactate leads to TME acidosis. The acidic TME makes cancer cells a strong survival advantage and drug resistance. d. DNA damage repair is a classic mechanism for drug resistant. e. Drug resistant-cancer cells are often accompanied by downregulation of intracellular apoptotic proteins or up-regulation of anti-apoptotic proteins. f. Targeting therapy of CSCs by inhibiting EMT has become an effective way to anticancer and prevent drug resistance.

The mechanism of drug resistance is complex, the currently known mechanisms of drug resistance include drug efflux (98), mutation of drug target (99, 100), alterations in drug metabolism (101), DNA damage repair (102, 103), alterations of energy programming, cancer stem cells and epigenetic changes (104, 105). Most of these processes were regulated by exosomal miRNA (Figure 2). Different treatments have been developed to circumvent these resistance mechanisms (106–112). In this section we associate several mechanisms of drug resistance with miRNAs.

### Drug Efflux and Metabolism in Cancers

Drug resistance is always accompanied by the dysfunction of pharmacokinetic factors, that are absorption, distribution, metabolism and elimination (ADME) of drugs. Exosomal miRNA participate drug resistance by interfering drug efflux and metabolism as well.

The excessive drug efflux is a classic mechanism of drug resistance. The human ATP-binding cassette (ABC) transporter superfamily is closely associated with the excessive efflux of drug. In the ABC transporter superfamily, several ATP-driven efflux transporters are the classic regulator of drug efflux: ABCB1 (P-gp/MDR1), ABCC1 (MRP1), ABCG2 (BCRP), ABCC2, MDR4 and MDR5 (113–118). The efficacy of drugs is closely related to the concentration of the drug inside the cells. In drug resistant-tumor cells, overexpression of these ABC transporters pumps anticancer drug out of cells, decreasing the concentration of drugs.

Tumor-derived exosomal miRNA cargo regulates the expression of ABC transporters and facilitates drug resistance in tumor cells. ABCB1 is one of the ABC transporters, some researchers reported that ABCB1 enriched in microvesicles and exosomes shed by drug-resistant cells (119). These EVs transfer ABCB1 to drug-sensitive cells, making the recipient cells express functional ABCB1 and acquiring drug resistance. However, the half-life of ABCB1 is shorter than 24 h and the transfer of ABCB1 is unstable (120). The resistant mechanism of drug-sensitive cells cannot be merely explained by the transfer of ABCB1. Sousa conjectured in his review that ABCB1 may co-transport with miRNA so that ABCB1 can be expressed stably for a long time (121). After that, exosomal miRNA modulates transcripts in recipient cells to acquire resistance phenotype (122). For example, exosomal miR-1246 secreted by ovarian cancer (OC) cells inhibits the expression of Cav1 and upregulates ABCB1 expression to induce tumor-promoting phenotype and drug resistance. Based on the preclinical experiments *in vivo*, miR-1246 inhibitor treatment in combination with chemotherapy shows great potential in the treatment of OC (12). Exosomal miRNA is a double-edged sword in the occurrence and development of drug resistance. Some miRNAs have positive effects in drug resistance, while some can enhance the chemosensitivity of cancer cells. Liu et al. found that exosome-transmitted miR-128-3p down-regulates the expression of MDR5, decreasing oxaliplatin efflux and improving chemosensitivity of oxaliplatin-resist cells in colorectal cancer (123).

The activation of drug is related to the corresponding enzymes in body. The prodrug is activated by enzyme action, or the drug is metabolized into an inactive form due to some enzymes *in vivo*. For example, gemcitabine is metabolized by deoxycytidine kinase (dCK) and incorporate with nucleosides in DNA and RNA, preventing DNA from replicating properly. Cytidine deaminase (CDA) is an enzyme that metabolizes gemcitabine to become an inactive form. When the tumor emerges drug resistance, it is often accompanied by the inactivation of dCK or activation of CDA, leading to gemcitabine degradation or inactivation and eventually causing drug resistance (78, 124, 125). Exosomal miRNA is involved in drug metabolism. In pancreatic adenocarcinoma, tumor associated macrophages secrete exosomes that transferring miR-365 to induce gemcitabine-resistance (79). The specific mechanism is that macrophage-derived exosomes (MDE) transfer miR-365 into pancreatic ductal adenocarcinoma (PDAC) cells. Once miR-365 entered PDAC cells, the concentration of triphosphate-nucleotide (NTP) is increasing. NTP can compete with phosphorylated gemcitabine for DNA incorporation, so that it prevents activation of gemcitabine. Moreover, exosomal miR-365 upregulates the expression of CDA and promotes the inactivation of gemcitabine leading to gemcitabine resistance (79).

### Metabolic Reprogramming and TME Acidosis

Energy reprogramming has been accepted as a hallmark of cancer (126). In order to maintain survival, proliferation and dissemination, cancer cells need to reprogram their metabolism to ensure the increasing energy demand (127–129). Mitochondrial oxidative phosphorylation (OXPHOS) and glycolysis are two major metabolic pathways to generate adenosine triphosphate (ATP) to support physiological activities in our daily life. A common characteristic in primary and metastatic cancer is the upregulation of glycolysis (130). Glycolysis usually occurs in an anoxic condition. However, even in aerobic conditions, cancer cells undergo aerobic glycolysis by reprogramming the glucose metabolism and glycolysis is still widespread in TME. This phenomenon is called Warburg Effect. An important reason of this effect is that during glycolysis, glucose is metabolized into pyruvate and lactate. In cancer cells, excessive production of lactate leads to TME acidosis (131). The acidic TME largely contributes to the immunologic escape, because the decrease in extracellular pH leads to the reduction of cytotoxic T- cell function, thus the cancer cells can acquire a strong survival advantage which promotes cancer metastasis, invasion and drug resistance (132–134).

Regulation of glycolysis is one of the ways to inhibit cancer drug resistance (134). The GLUT family is closely related to glucose transport into cells. GLUT1, one of the family members in GLUT, is upregulated in many malignant tumors (135). The upregulation of GLUT1 is associated with mammalian target of rapamycin (mTOR) and the activation of mTOR increases glycolysis and promotes drug resistance (132). The decreased expression of miR-100 is involved in drug resistance in several cancer. mTOR is a target gene of miR-100-5p which binds to the 3′UTR directly and decreases the expression of mTOR and enhances chemo-sensitive of cancer cells. Qin et al. (136) indicated in their study that the expressing of miR-100-5p is not only related to the cell itself, but also to the extracellular microenvironment. Exosome as a messenger for intracellular communication, the concentration of miR-100-5p in exosomes is reflected the content in surrounding microenvironment. The downregulation of miR-100-5p in microenvironment leads to cisplatin resistance in lung cancer cells (136). In addition, TP53INP1 is also a stress protein, which has been indicated to play a tumor suppressive role by regulating metabolic homeostasis (137). Fang et al. showed that CAF derives exosomal miR-106b, which promotes gemcitabine resistance by directly targeting TP53INP1 (138).

### DNA Damage Repair

As a target of anticancer drugs, DNA damage induces cancer cell death. Genotoxic agents are designed for damaging DNA or preventing the synthesis of new DNA to inhibit cell proliferation. Genotoxic agents are classified as direct damage, such as cisplatin; and indirect damage, such as topoisomerase inhibitors (24). However, in addition to cell death, DNA damage response (DDR) includes the DNA damage repair (139).

DNA damage repair is originally a way to maintain genomic stability in cells. However, DNA damage repair has also been found to be a resistance mechanism because of the widespread use of genotoxic agents (140). DNA repair mechanisms can be briefly divided into the following four categories: (a) Nucleotide excision repair (NER): NER works in a way that is suitable for repairing bulky DNA lesions by using DNA ligase to attach repair patch to the damage DNA regions, which is associated with platinum agent resistance. (b) Base excision repair (BER): BER works through repairing a small number of bases and performing some modification, such as alkylation and oxidative lesions. This repair mechanism is related to the resistance of genotoxic agents nitrosoureas. (c) Mismatch repair (MMR): MMR participates in the modification of oxidation and methylation by bypassing the lesions to replicate. (d) DNA double-strand break repair: Double-strand break (DSB): DSB is the most toxic form of DNA damage. Two main repair pathways of DSB are non-homologous end joining (NHEJ) and homologous recombination (HR) (139, 141). Briefly, these two repair pathways have their own characteristics. NHEJ is more rapid, while HR is more complex and accurate. This mechanism is applicable to the damage induced by topoisomerase inhibitors, temozolomide (TMZ) and some alkylating agents (142).

Exosomal miRNA is a regulator to inhibit DNA damage repair. XRCC4 is a major participator of NHEJ, which forms a heterodimer with DNA ligase IV and covalently joins the broken DNA (143). There have been reports of XRCC4 linked to TMZ resistance in earlier years. XRCC4 is a direct target of miR-151a, the low expression of which leads to the upregulation of XRCC4 and triggers the DNA repair that makes cell resistant to TMZ. To investigate the effects of exosomal miR-151a on cancer cells, researchers incubated glioblastoma multiforme (GBM) receptor cells with exosomes secreted by TMZ-resistant cells and TMZ-sensitive cells. The result shows that GBM receptor cells co-cultured with TMZ-resistant exosomes have stronger resistance to TMZ. However, when researchers restore miR-151a in TMZ-resistant exosomes, the TMZ resistance of GBM recipient cells is significantly decreases (144). This study shows that exosomes have the ability to transfer chemoresistance to sensitive cancer cells and exosomal miR-151a has the potential to become a prognostic factor in GBM treatment.

### Deregulation of Apoptosis

Resisting cell death is a characteristic of cancers, which leads the unlimited proliferation of cancer cells and the development of drug resistance (145, 146). Drug resistant-cancer cells are often accompanied by downregulation of intracellular apoptotic proteins or up-regulation of anti-apoptotic proteins.

Exosome secreted by drug-resistant cells can transmit the resistance to neighboring cells. Zhang et al. (147) indicated that exosomal miR-214 mediates gefitinib resistance in non-small cell lung cancer (NSCLC). Compared with sensitive cancer cells, the miR-214 in exosomes secreted by gefitinib resistant-cells is significantly increased. Gefitinib resistant-cells secreted exosomal miR-214 could confer gefitinib resistance in NSCLC by suppressing cell apoptosis (147). In addition to cancer cells, exosomes secreted by stroma cells also act on resistant targets by transferring miRNA, making cancer cells to acquire drug resistance. Paclitaxel is a common agent for the treatment of ovarian cancer. However, the efficacy of paclitaxel treatment is greatly reduced if the ovarian cancer cells develop resistance to paclitaxel. By using sequencing technology, Au Yeung et al. (148) identified that miR-21 isomiRNAs have higher expression level in the exosomes of cancer-associated adipocytes (CAAs) and CAFs than in those from ovarian cancer cells. After exosomal miR-21 transship to ovarian cancer cells, miR-21 binds to apoptotic protease activating factor 1 (APAF1) and the expression of APAF1 is downregulated (148). APAF1 combined with cytochrome c (Cyt-c) and dATP to form apoptosomes, increasing caspase-9 and caspase-3, leading to massive mitochondrial damage and finally inducing cell apoptosis (149). Therefore, the decrease of APAF1 has the ability to suppress apoptosis and eventually cause drug resistance in cancer cells. This result showed that in omental tumor microenvironment, cancer cells have a negative effect on neighboring stromal-derived exosomal miR-21 and acquire malignant phenotype, including drug resistance (148). Moreover, exosomal miR-196a derived from CAFs confers cisplatin resistance in head and neck cancer (HNC). In order to explore the mechanism of exosomal miR-196a in HNC cells, Qin et al. (150) used miRecords algorithm and finally found the target of exosomal miR-196a: CDKN1B and ING5. CDKN1B and ING5 exhibit different functions in miR-196a-mediated cisplatin resistance. ING5 gene is a major gene to regulate apoptosis. Therefore, they proposed that exosomal miR-196a promote cisplatin-resistance in HNC cells by suppressing apoptosis of cancer cells (150).

### Epithelial-to-Mesenchymal Transition (EMT) and Cancer Stem Cells (CSCs)

In the process of cancer growth, genetic and non-genetic factors induce biological heterogeneity, resulting in phenotypic difference of tumors. The phenotypic diversity of malignant cancers is considered as a significant driver that induces drug resistance.

The cancer stem cells (CSCs) concept provides a good explanation for the association between heterogeneity and the resistance of cancer cells. Because of the renewal properties and genomic instability, CSCs are closely related to the proliferation, metastasis, and recurrence of cancer (151). Epigenetic regulation has a great contribution to the behaviors of cancer cells. Epigenetic differences between CSCs and non-CSCs have a great possibility that caused by epithelial-to-mesenchymal transition (EMT) (152). When epithelial cells transform into mesenchymal cells, cancer cells acquire the properties of migration and invasion and even drug resistance (153–155). What's interesting lies on the study that shows that EMT only occurs in tumors with CSCs (156–158). In tumor microenvironment, CSCs comprise a small proportion of total cells in tumor, most of the cancer cells are non-CSCs (157). However, the traditional cancer treatment merely kills most of the non-CSCs and the CSCs are retained. These residual CSCs eventually induce tumor recurrence and drug resistance through differentiation (159–162).

In recent years, targeting therapy of CSCs by inhibiting EMT has become an effective way to treat cancers and prevent drug resistance (163–167). EMT is an effective target to affect drug resistance. Exosomal miR-32-5p is proved to induce multidrug resistance in hepatocellular carcinoma via the PI3K/AKT pathway to promote EMT and angiogenesis (168). CSCs themselves also secret exosomes to induce drug resistance. MiR-155 is a classic and multifunctional modulating miRNA which is overexpressed in multiple malignant cancers (169). Santos et al. (170) carried out a study which supports a putative mechanism of exosomal miRNA transmission between cancer cells: miR-155 is enriched in exosomes secreted by CSCs and drug resistant cells. In addition, they observed the downregulation of E-Cadherin (E-Cad) and upregulation of mesenchymal biomarkers, which demonstrated that CSCs and drug resistant cells have the ability to trigger the EMT process in recipient cells by transferring exosomal miR-155 and eventually lead to the recipient cells possess resistance (170). In pancreatic cancer cells, the gemcitabine-resistant CSCs can secret miR-210 enriched exosomes. Gemcitabine-resistant CSCs enhance drug resistant by transferring exosomal miR-210 to gemcitabine-sensitive cells (171).

During these years, more and more studies have revealed that different types of cells secrete exosomal miRNA in tumor microenvironment and participate in the process of drug resistance. The drug resistant mechanism of exosomal miRNA on several common anticancer chemotherapeutic agents and molecular targeted agents are summarized in Table 2.

**Table 2:** Summary of common anticancer drugs and exosomal miRNA involved in drug resistance.

**Anticancer drug type**	**Agents**	**Cancer type**	**Exosomal miRNA**	**Resistance mechanism**	**References**
Antimetabolites	5-FU	Hepatocellular Carcinoma	miR-32-5p	Promote angiogenesis and EMT	(168)
	Gemcitabine	Pancreatic Cancer	miR-365	Prevent gemcitabine activation and promote gemcitabine inactivation	(79)
		Non-small Cell Lung Cancer	miR-222-3p	Directly target the promoter of SOCS3 to transfer malignant phenotypic trait	(63)
		Pancreatic Cancer	miR-210	Inhibit GEM-induced cell cycle arrest, antagonize GEM-induced apoptosis, and promote tube formation and cell migration	(171)
		Pancreatic Cancer	miR-155	Suppressing the key gemcitabine-metabolizing enzyme, DCK	(78)
		Pancreatic Cancer	miR-106b	Promote GEM resistance of cancer cells by directly targeting TP53INP1	(138)
Platinum compounds	Cisplatin	Head and Neck Cancer	miR-196a	Exosomal miR-196a derives from CAFs binds novel targets, CDKN1B and ING5, to endow HNC cells with cisplatin resistance	(150)
		Gastric Cancer	miR-21	Suppress cell apoptosis and enhance activation of PI3K/AKT signaling pathway by down-regulation of PTEN	(16)
		Lung Cancer	miR-100-5p	Exosomes confer recipient cells' resistance to cisplatin in an exosomal miR-100-5p-dependent manner with mTOR as its potential target both *in vitro* and *in vivo*	(136)
		Non-small Cell Lung Cancer	miR-425-3p	Exosomal miR-425-3p facilitated autophagic activation in the recipient cells by targeting AKT1, eventually leading to chemoresistance	(172)
	Carboplatin	Breast Cancer	miR-222/223	Exosomal miR-222/223 promote quiescence in a subset of cancer cells and confers drug resistance	(173)
	Oxaliplatin	Colorectal Cancer	miR-128-3p	miR-128-3p suppress EMT and increased intracellular oxaliplatin accumulation	(123)
		Colorectal Cancer	miR-46146	Directly target PDCD10 and induce oxaliplatin chemoresistance	(174)
Topoisomerase inhibitor	Doxorubicin	Gastric Cancer	miR-501	Downregulate BLID, inactivate caspase-9/-3 and phosphorylate Akt	(175)
Microtubule poisons	Paclitaxel	Ovarian Cancer	miR-21	Target APAF1 and confer chemoresistance	(148)
		Ovarian Cancer	miR-1246	Target Cav1/p-gp/M2-type Macrophage Axis	(12)
		Gastric Cancer	miR-155-5p	Induce EMT and chemoresistant phenotypes	(176)
Molecular targets agents	Imatinib	Chronic Myeloid Leukemia	miR-365	Inhibit expression of pro-apoptosis protein in sensitive CML cells	(177)
	Trastuzumab	Breast Cancer	miR-567	Suppress autophagy and reverse chemoresistance by targeting ATG5	(178)
	Gefitinib	Non-small Cell Lung Cancer	miR-214	–	(147)

## Conclusions

Drug resistance is an eternal topic in cancer treatment. In this article, we discussed the role of exosomal miRNA in different mechanisms of drug resistance. Some of them act as “communicators” and some of them “biomarkers” that facilitate communication between cancer cells with other cancer cells or cancer cells with tumor microenvironment, enriching the knowledge background about the diagnosis of cancer. However, drug resistance in cancer is not caused by only one or several mechanisms, it is the combined action of the intrinsic (such as mutation) and extrinsic (such as drug inactivation) factors. Although progress has been made in suppressing the emergence of drug resistance, there is still a long way to go to eradicate the problem of drug resistance. Nevertheless, the knowledge of exosomal miRNA will provide some clues to help exploring the secret of cancer drug resistance.

## Author Contributions

QG, QW, and JZ conceived the review. QG, YL, and CS searched the literature and drafted the manuscript. YaY, RA, and HC critically appraised the literature. YiY, HW, and CS edited the manuscript. All authors approved the final version of the manuscript.

### Conflict of Interest

The authors declare that the research was conducted in the absence of any commercial or financial relationships that could be construed as a potential conflict of interest.
